# A Pyroptosis-Related Signature Predicts Overall Survival and Immunotherapy Responses in Lung Adenocarcinoma

**DOI:** 10.3389/fgene.2022.891301

**Published:** 2022-06-20

**Authors:** Kaibin Zhu, An Yan, Fucheng Zhou, Su Zhao, Jinfeng Ning, Lei Yao, Desi Shang, Lantao Chen

**Affiliations:** ^1^ Department of Thoracic Surgery, Harbin Medical University Cancer Hospital, Harbin, China; ^2^ Department of Thoracic Oncology, Harbin Medical University Cancer Hospital, Harbin, China; ^3^ College of Bioinformatics Science and Technology, Harbin Medical University, Harbin, China

**Keywords:** glycosyltransferase, lung adenocarcinoma, prognosis, gene signature, immunotherapy

## Abstract

**Background:** Lung adenocarcinoma (LUAD) is a highly malignant cancer with a bleak prognosis. Pyroptosis is crucial in LUAD. The present study investigated the prognostic value of a pyroptosis-related signature in LUAD.

**Methods:** LUAD’s genomic data were downloaded from TCGA and GEO databases. K-means clustering was used to classify the data based on pyroptosis-related genes (PRGs). The features of tumor microenvironment were compared between the two subtypes. Differentially expressed genes (DEGs) were identified between the two subtypes, and functional enrichment and module analysis were carried out. LASSO Cox regression was used to build a prognostic model. Its prognostic value was assessed.

**Results:** In LUAD, genetic and transcriptional changes in PRGs were found. A total of 30 PRGs were found to be differentially expressed in LUAD tissues. Based on PRGs, LUAD patients were divided into two subgroups. Subtype 1 has a higher overall survival rate than subtype 2. The tumor microenvironment characteristics of the two subtypes differed significantly. Compared to subtype 1, subtype 2 had strong immunological infiltration. Between the two groups, 719 DEGs were discovered. WGCNA used these DEGs to build a co-expression network. The network modules were analyzed. A prognostic model based on seven genes was developed, including FOSL1, KRT6A, GPR133, TMPRSS2, PRDM16, SFTPB, and SFTA3. The developed model was linked to overall survival and response to immunotherapy in patients with LUAD.

**Conclusion:** In LUAD, a pyroptosis-related signature was developed to predict overall survival and treatment responses to immunotherapy.

## Introduction

Lung cancer is a worldwide public health problem (Bray et al., 2018). The most common subtype of lung cancer is lung adenocarcinoma (LUAD) (Cheng et al., 2016). Despite advancements in lung cancer treatment, patients have a 5-year survival rate of less than 20% (Dixon et al., 2014). The clinical application of immunotherapies enhanced lung cancer therapy (Memmott et al., 2021). However, some lung cancer patients do not respond to immunotherapies (Peters et al., 2019). As a result, it is critical to investigate markers for predicting the lung cancer prognosis.

Pyroptosis is a type of programmed cell death that results in the release of pro-inflammatory cytokines (Liu et al., 2021). Pyroptosis is primarily triggered by the cleavage of gasdermin D (GSDMD) and the activation of NLRP3/caspase-1 (Schneider et al., 2017; Wei et al., 2020). Pyroptosis has been linked to various cancers, including liver cancer, cervical cancer, and breast cancer. (A et al., 2014; Chu et al., 2016; Chen et al., 2021). Ye et al. (2021) found that PRGs play a significant role in tumor immunity. The defined pyroptosis-related signature might be utilized to predict the prognosis of ovarian cancer. In lung cancer patients' alveolar macrophages, NLRP3/caspase-1 inflammasome is suppressed (Lasithiotaki et al., 2018).

Furthermore, the activation of pyroptosis has an inhibitive effect on lung cancer. Polyphyllin VI has an anticancer action associated with pyroptosis activation (Teng et al., 2020). Resibufogenin may suppress lung cancer development and metastasis by triggering pyroptosis (Yin et al., 2021). GSDMD downregulation may limit lung cancer cell growth *via* the EGFR/Akt signaling pathway. Patients with LUAD who had less GSDMD expression had a better prognosis (Gao et al., 2018). As a result, PRGs may have prognostic and therapeutic potential in LUAD management.

We investigated the role of pyroptosis in the prognosis of LUAD, utilizing a pyroptosis-related signature in this study. The established prognostic model might predict LUAD patients' overall survival (OS) and responses to treatment. This study would promote the rationale use of immunotherapy in LUAD.

## Materials and Methods

### Data Sources

The Cancer Genome Atlas (TCGA) genomic data for LUAD samples were obtained from the Genomic Data Commons. Gene Expression Omnibus (GEO) was used to download gene expression microarrays of LUAD samples (GSE31210) and non-small cell lung cancer (NSCLC) samples (GSE37745 and GSE50081) and lung cancer (GSE30219). The Robust Multichip Average (RMA) method and R package “affy” normalized GSE37745 gene expression data. Detailed information of the cohorts is presented in Supplementary Tables S1, S2.

IMvigor210 was a single-arm phase Ⅱ study that looked into an anti-PD-L1 agent (atezolizumab) in patients with metastatic urothelial carcinoma (mUCC) (NCT02108652 and NCT02951767) (Mariathasan et al., 2018). The R package “IMvigor210CoreBiologies” obtained all the expression and clinical data from the IMvigor210 trials. GEO provided RNA-seq data for a total of 27 advanced NSCLC patients who were treated with anti-PD-1/PD-L1 (GSE135222).

### Variation and Interactions of Pyroptosis-Related Genes

A total of 47 PRGs were obtained from the study of Song et al. (2021). The R package “maftools” was used to demonstrate PRG mutation. The R package “ggpubr” was used to visualize the copy number variation (CNV) information of PRGs. The R package “limma” was used to examine the differential expression of PRGs in tumor samples.

The Pathway Commons database was used to find PRG protein–protein interactions. Pearson correlation was used to examine the co-expression status of PRGs (Supplementary Table S3). Cytoscape software was utilized to visualize the correlation network.

### Identification of Pyroptosis-Related Subtypes

Based on the pyroptosis genes and R package “pheatmap,” K-means clustering was used to determine the pyroptosis-related subtypes (subtypes 1 and 2). The Kaplan–Meier survival analysis was performed to analyze patient differences between the two subtypes in conjunction with the log-rank test. The difference between two subtypes based on the PRG expression was investigated using principal component analysis (PCA).

### Distinction of Cancer Therapeutic Signatures Between Subtypes

We obtained 25 cancer treatment-predicted signature sets from various publications (Sweis et al., 2016; Ayers et al., 2017; Mariathasan et al., 2018; Kamoun et al., 2020). The R package “GSVA” was used to calculate the therapeutic signature gene set enrichment score using gene set variation analysis (GSVA). Detailed information of 25 cancer treatment-predicted signature sets is listed in Supplementary Table S4. The one-sided Wilcoxon rank-sum test was used to analyze the differences in the therapeutic enrichment scores between subtypes.

### Characteristics of the Tumor Microenvironment

The range of infiltration of 22 immune cells in TCGA LUAD samples was inferred by the CIBERSORT (Cell-type Identification by Estimating Relative Subsets of RNA Transcripts) method (Newman et al., 2015). CIBERSORT can compute the abundances of specific cell types in a mixed sample based on the bulk expression. In addition, the ESTIMATE (Estimation of STromal and Immune Cells in MAlignant Tumor Tissues Using Expression Data) method was used to calculate the abundances of immune cells by the R package “estimate.” We focused on the mRNA expression of five immune checkpoints: PD-1, PD-L1, CTLA4, CD47, and BTLA. The one-sided Wilcoxon rank-sum test was utilized to analyze the differences between subtypes.

### Functional Analysis for Subtypes

The R package “limma” discovered 719 differentially expressed genes (DEGs) between two subtypes with |log2FC| > 0.5 and *p* < 0.001. A web-based program, Metascape, was used to perform the enrichment analysis on 719 DEGs using ontology sources such as KEGG Pathway, GO, Reactome, and other canonical pathways (Supplementary Table S5). Then, a selection of enriched terms with a similarity greater than 0.3 was chosen and shown as a network plot.

### Identification of a Key Module

WGCNA (weighted gene co-expression network analysis) is a data reduction method and an unsupervised classification method (Langfelder and Horvath, 2008; Langfelder and Horvath, 2012). The co-expression network was built using the Sangerbox 3.0 tool and DEG expression profile. Module–trait association analysis was used to determine which co-expression module was the most relevant to the clinical features. The genes were clustered, and a heatmap was created to illustrate the relationship between modules and phenotype.

### Construction of a Pyroptosis Subtype-Related Prognostic Model

The least absolute shrinkage and selection operator (LASSO) approach and Cox regression model were employed to screen the prognostic genes in the key module. One standard error (SE) over the minimum threshold was chosen. The R package “glmnet” managed the entire process. Finally, a seven-gene risk score formula was developed, and multivariate Cox regression coefficients were computed using the R package “survival”: Pyroptosis subtype-related risk score (PSR_score) = (exp Gene1 * coef Gene1) + (exp Gene2 * coef Gene2) + … +(exp Gene7* coef Gene7).

### Survival Analysis

Patients were classified based on the median of their PSR_score. The R package “survival” used the log-rank test to compare the survival times of patients with high PSR_score and patients with low PSR_score. Furthermore, stratified analysis was performed to determine the protective effect of PSR_score based on the T stage, N stage, M stage, and tumor stage. Chi-square tests were used to examine the connections between the PRG score and clinical factors such as age, gender, T stage, N stage, and M stage. The data were presented using Kaplan–Meier graphs (Supplementary Table S1, S2).

### Statistical Analysis

The one-sided Wilcoxon rank-sum test was used to determine the difference between the two subtypes or high- and low-PSR_score groups. R version 4.1.2 was used for all statistical studies. *p* < 0.05 was considered statistically significant.

## Results

### Genetic and Transcriptional Alterations of Pyroptosis Genes in Lung Adenocarcinoma

**FIGURE 1:**
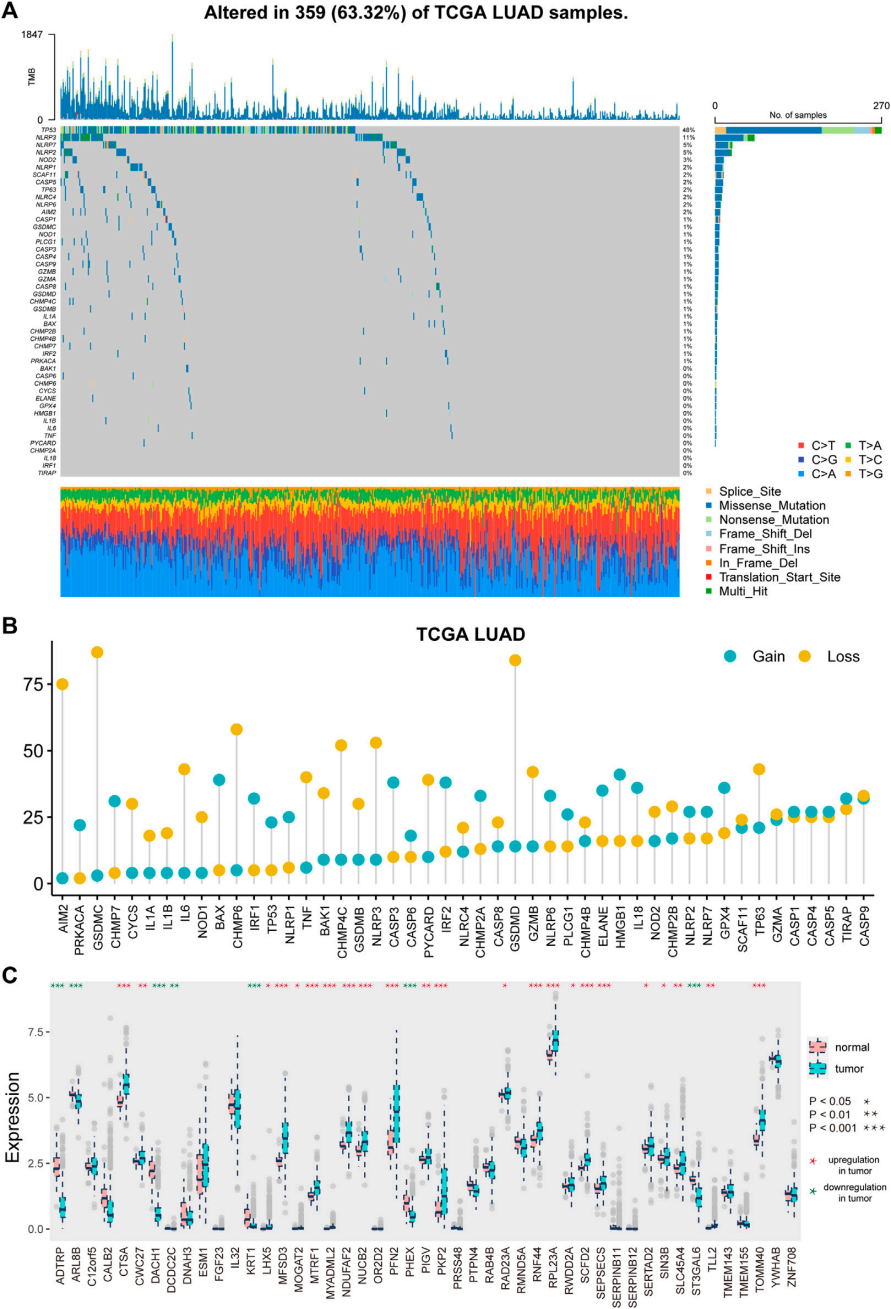
Genetic and transcriptional alterations of pyroptosis-related genes in LUAD. **(A)** Mutation frequencies of pyroptosis-related genes in LUAD patients of TCGA cohort. **(B)** Frequencies of CNV gain and loss of pyroptosis-related genes in LUAD patients. **(C)** Expression distributions of pyroptosis-related genes between tumor and normal samples.

Supplementary Figure S1 depicts the analytical process used in this study. We first explored the landscape of variation in PRGs in the genome and transcriptome. A relatively high mutation frequency of PRGs was observed in LUAD (Figure 1A). TP53 exhibited the highest mutation frequency (55%), followed by NLRP3, NLRP7, and NLRP2. Then, we looked at the link between TP53 mutation and PRG expression. CHMP7, IRF2, CASP4, ELANE, BAX, and TIRAP were all downregulated in TP53 mutation samples (Supplementary Figure S2, *p* < 0.1). Following that, we investigated the CNV landscape of PRGs in LUAD (Figure 1B). Copy number amplification was common in HMGB1, BAX, CASP3, IRF2, IL18, and GPX4, whereas copy number deletion was common in GSDMC, GSDMD, AIM2, and CHMP6.

Furthermore, we investigated the difference in PRG expression levels between tumor and normal tissues (Figure 1C). A total of 30 (63.83%) PRGs showed differential expression (*p* < 0.05), with 23 genes showing substantial upregulation and seven showing significant downregulation in tumor samples.

### Identification of Pyroptosis-Related Subtypes

**FIGURE 2:**
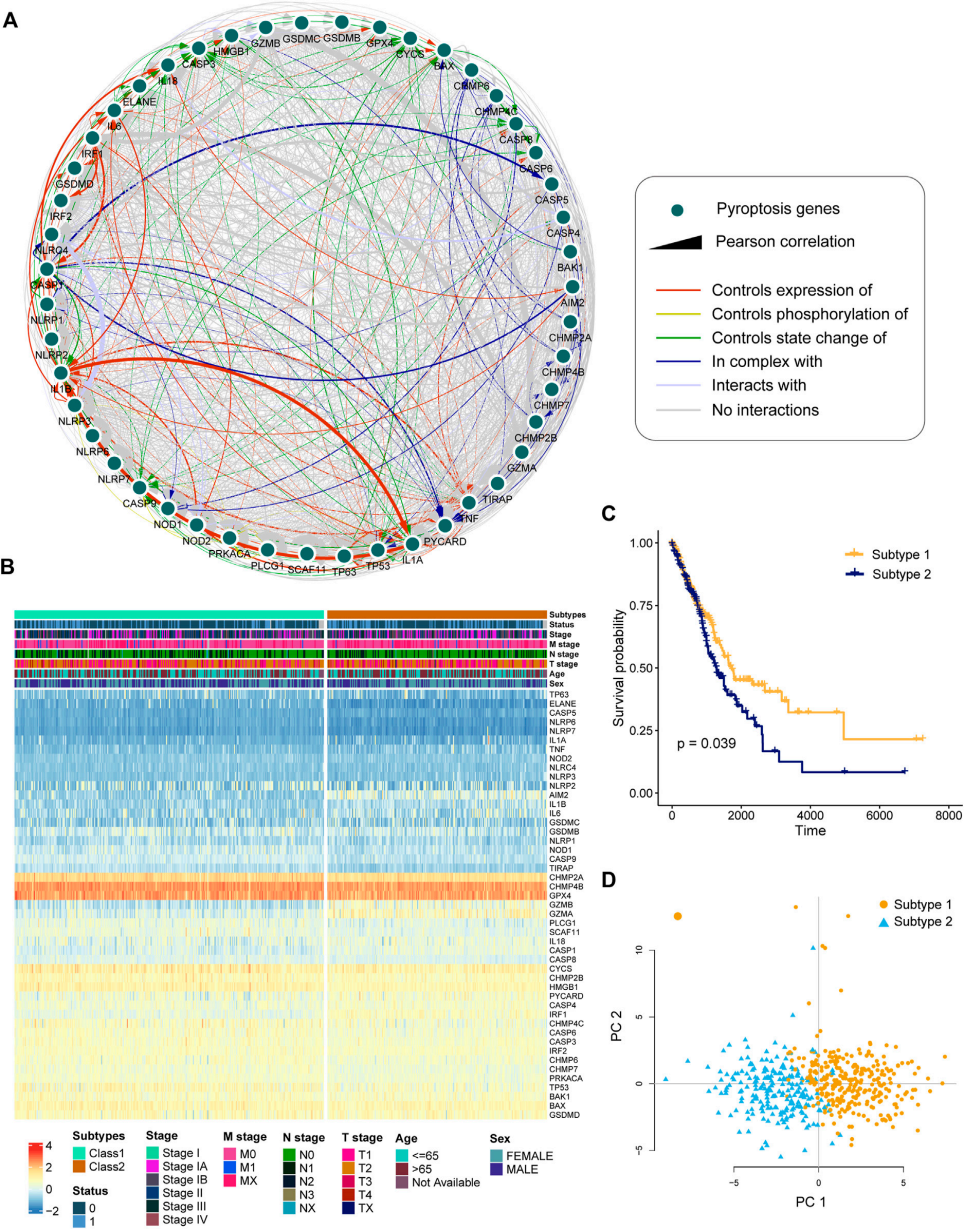
Identification of pyroptosis-related subtypes by clustering. **(A)** Interactions and co-expression among pyroptosis-related genes in LUAD. The colored edges represent protein–protein interactions, with the line thickness indicating the strength of the correlation between pyroptosis-related genes. **(B)** Two heterogeneous subtypes (subtype 1 and subtype 2) were identified according to unsupervised K-means clustering. **(C)** Kaplan–Meier curves of OS between subtype 1 and subtype 2. **(D)** PCA analysis demonstrating a remarkable difference in expression of pyroptosis-related genes between the two subtypes.

We built an interaction network to investigate the relationship between PRGs (Figure 2A). The color of the edges indicated the five types of protein–protein interactions, and the thickness of the edges indicated the level of co-expression between PRGs, as determined by Pearson correlation (Supplementary Table S3). The network showed a strong relationship between PRGs.

To investigate the heterogeneous features of LUAD further, a K-means clustering algorithm was used to categorize patients based on PRG expression profiles. Patients with LUAD were classified into two subtypes (Figure 2B). Survival analysis revealed that subtype 1 had a considerably greater overall survival than subtype 2 (Figure 2C, *p* = 0.039, log-rank test). According to principal component analysis (PCA), LUAD patients had unique PRG expression patterns between two subtypes (Figure 2D).

### Characteristics of the Tumor Microenvironment and Therapeutic Evaluation in Distinct Subtypes

**FIGURE 3:**
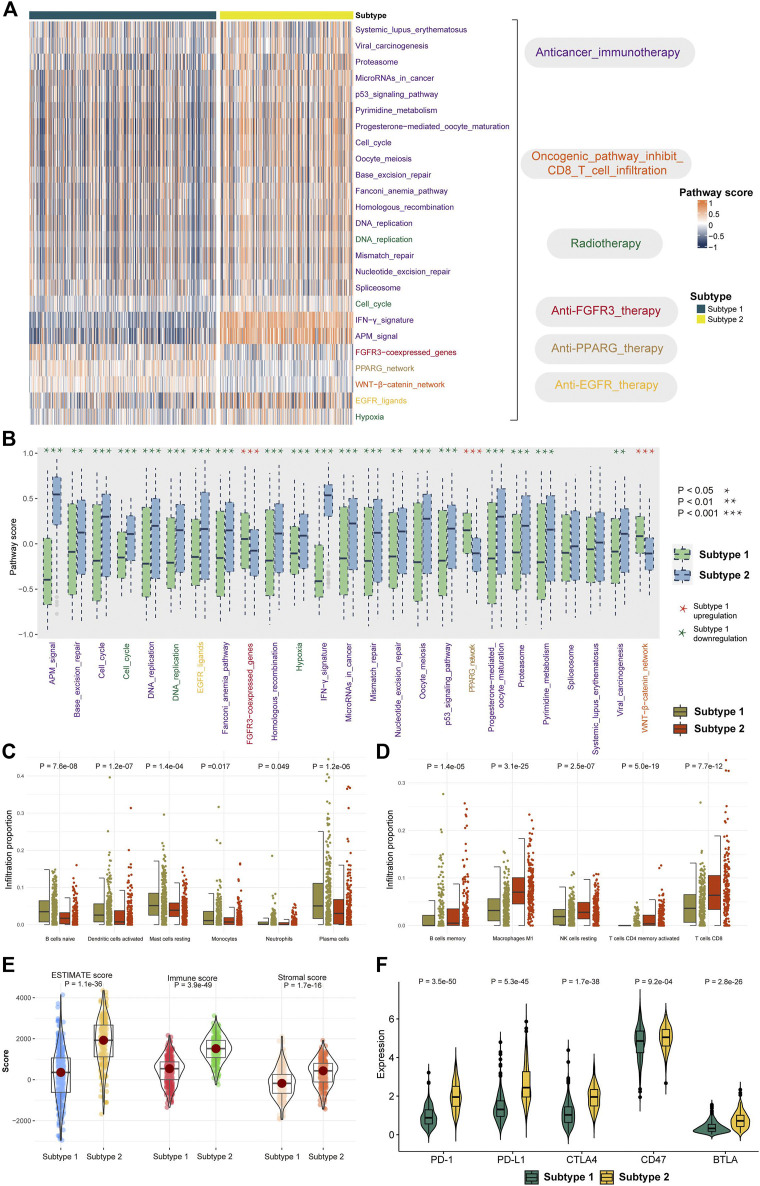
Distinction of therapeutic signature and TME between the subtypes. **(A)** Heatmap showed the GSVA score of 25 therapeutic signature gene sets in TCGA LUAD samples. The therapeutic signature gene sets belong to six categories. **(B)** Distribution of therapeutic signature score between two subtypes. **(C–D)** Abundance of infiltrating immune cell types in two subtypes. **(E)** Distribution of the ESTIMATE score in two subtypes. **(F)** Expression levels of five checkpoints in two subtypes.

The therapeutic differentiation between the subtypes was investigated, and the GSVA approach was utilized to determine the score of 25 therapeutic signature sets in TCGA LUAD data (Figure 3A). A total of 23 (92%) therapeutic signatures differed significantly between the two subtypes, with 20 therapeutic signature scores in subtype 2 significantly higher than those in subtype 1 and three therapeutic signature ratings significantly lower (Figure 3B, *p* < 0.05). Patients in subtype 2 were found to be more amenable to treatment.

The differentiation of TME between two subtypes is then evaluated. According to the CIBERSORT algorithm, infiltration of “B cells naive,” “dendritic cells activated,” “mast cells resting,” “monocytes,” and “neutrophil plasma cells” were higher in subtype 1 than in subtype 2 (Figure 3C, *p* < 0.05). “B cells memory,” “macrophages M1,” “NK cells resting,”, “T cells CD4 memory activated,” and “T cells CD8” showed significantly lower infiltration in subtype 1 than in subtype 2 (Figure 3D, *p* < 0.05). Furthermore, we investigated the tumor purity differentiation across the subtypes, finding that the ESTIMATE score, stromal score, and immune score in subtype 1 were considerably lower than those in subtype 2 (Figure 3E, *p* < 0.05). Furthermore, we investigated the distinction between the subtypes in the ability to recognize tumor cells and execute immune responses. We looked at the differential expression of five immunological checkpoints and discovered that the expression of all the five immunological checkpoints was considerably greater in subtype 2 than that in subtype 1 (Figure 3F, *p* < 0.05). The result indicated that samples in subtype 2 had a higher level of immune infiltration.

### Analysis of Functional Differences Between Subtypes Based on Differentially Expressed Genes

**FIGURE 4:**
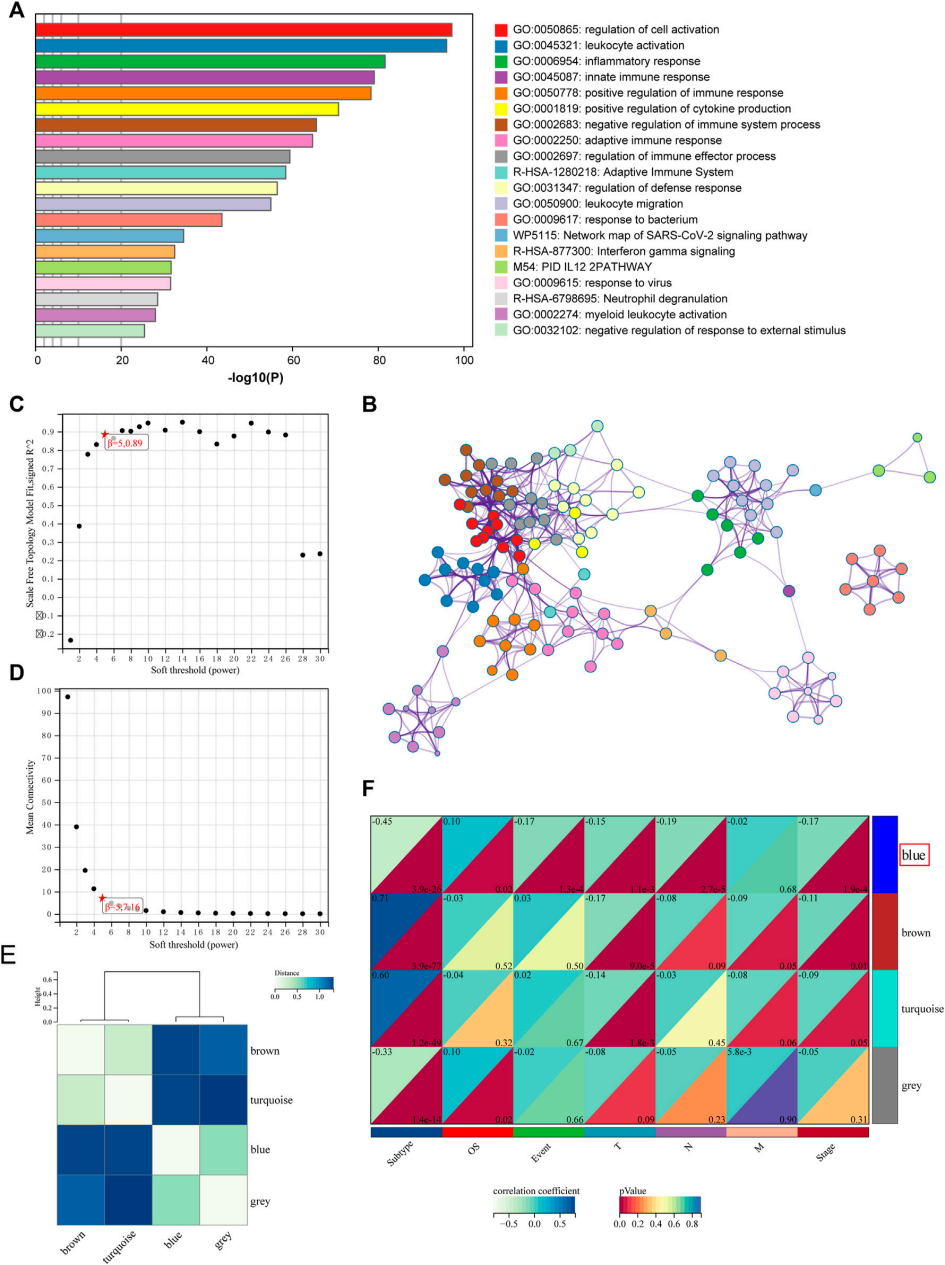
Functional analysis and identification of the co-expression module. **(A)** Pathway and process enrichment analysis has been conducted for DEGs that are identified between the subtypes. The graphical representation showed top 20 enrichments with *p* < 0.01. **(B)** Enrichment terms with a similarity > 0.3 are connected by edges. **(C–D)** Analysis of the scale-free fit index for various soft-thresholding powers and the mean connectivity for various soft-thresholding powers. **(E)** Clustering relationships among WGCNA modules. **(F)** Correlation between modules and clinical features. Blue represents a positive correlation, and white represents a negative correlation.

To investigate the potential biological activity of the subtypes, we detected DEGs between the two subtypes, and Metascape performed enrichment analysis on 719 DEGs (Figures 4A,B). The DEGs were found to be significantly enriched in a variety of immune-related pathways and processes, including “leukocyte activation,” “inflammatory response,” “innate immune response,” and “positive regulation of immune response” (Supplementary Table S5).

Then, WGCNA was used to build co-expressed networks based on the expression of 719 DEGs and identify important modules linked with clinical traits. The power value for modules was screened to ensure an average connection and high independence. The power value in this study was set at 5 as the soft-thresholding parameter to ensure a scale-free network (Figures 4C,D). In total, four modules have been identified (Figure 4E). The module–trait association analysis was used to discover co-expression modules that were highly relevant to clinical traits. Figure 4F depicts the relationship between modules and phenotype. Correlation analysis revealed that the blue module, which comprises 91 genes, was identified as a correlation between the prognosis and tumor stage. The top five highly enriched phrases for blue module genes were “secretion,” “cellular-modified amino acid metabolic process,” “epidermis development,” “NABA MATRISOME ASSOCIATED,” and “malignant pleural mesothelioma” (Supplementary Figure S3; Supplementary Table S5).

### Construction and Validation of the Prognostic PSR_score

**FIGURE 5:**
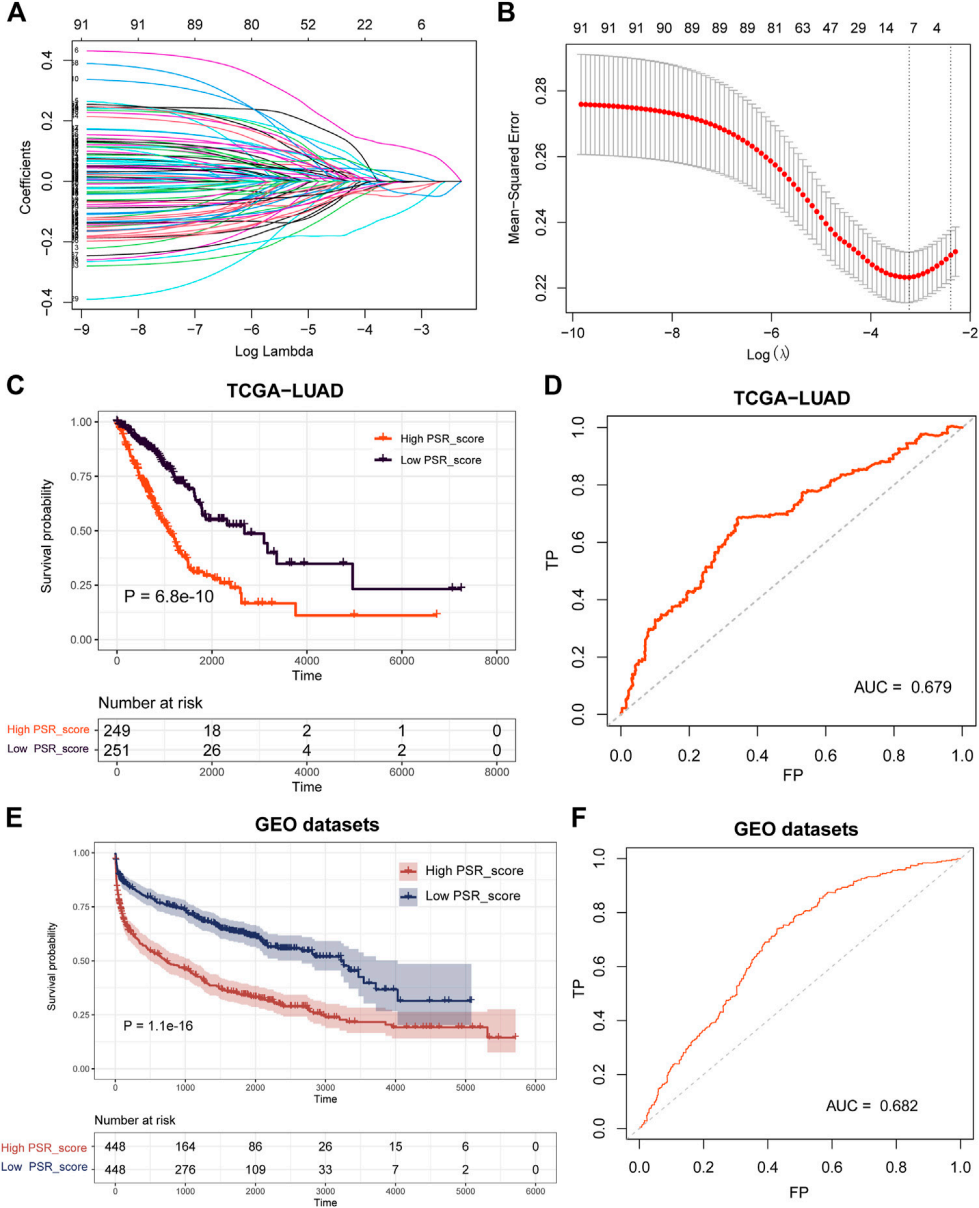
Construction and validation of the prognostic PSR_score by LASSO and COX regression analysis. **(A)** LASSO coefficient profiles of 91 blue co-expression module genes. **(B)** Cross-validation for tuning parameter selection in the LASSO model. **(C–D)** Log-rank test was employed to assess the difference in OS between high and low PSR_score samples in TCGA cohorts and ROC curve of the prognostic model. **(E–F)** Log-rank test was utilized to assess the difference in OS between high and low PSR_score samples in the integrated lung cancer cohorts and ROC curve of the prognostic model.

A model was built with seven pyroptosis subtype-related co-expression prognostic genes, FOSL1, KRT6A, GPR133, TMPRSS2, PRDM16, SFTPB, and SFTA3, to investigate the prognostic value of the selected subtype-related co-expression blue module genes (Figures 5A,B). Then, using the expression of seven genes, we established a predictive model according to the multivariate Cox proportional hazard model: PSR_score = (0.1072 * FOSL1 exp) + (0.09327 * KRT6A exp) + (−0.1144 * GPR133 exp) + (0.04062 * TMPRSS2 exp) + (−0.1238 * PRDM16 exp) + (−0.02503 * SFTPB exp) + (−0.04079 * SFTA3 exp).

The PSR_score of each patient in TCGA was calculated using the seven-gene-involved formula. The patients were divided into two groups using the median as the cutoff value: those with a high PSR_score and those with a low PSR_score. Patients with a high PSR_score had a substantially shorter life expectancy (Figure 5C, *p* = 6. 8e-10, log-rank test). The area under the curve (AUC) of the receiver operating characteristic (ROC) curve revealed that PSR_score correctly predicted mortality (Figure 5D, AUC = 0.679). We then investigated PSR_score’s ability to predict patient prognosis within clinicopathological subgroups. In most cancer stages, high PSR_score patients had a substantially worse OS than low PSR_score patients (Supplementary Figure S4, *p* < 0.05, log-rank test).

Following that, we validated the prognosis power of PSR_score in independent datasets. Survival analysis was carried out in four GEO lung cancer cohorts (GSE30219, GSE31210, GSE37745, and GSE50081), and the results revealed that a high PSR_score indicated a poor prognosis in all GEO datasets (Supplementary Figure S5, *p* < 0.1, log-rank test). We combined four GEO lung cancer cohorts into a big dataset to confirm the robustness of PSR_score. Similarly, patients with a high PSR_score had a significantly poor OS (Figure 5E, *p* = 1.1e-16, log-rank test), with an AUC of 0.682 (Figure 5F).

### Correlation of PSR_score and Immunotherapy

**FIGURE 6:**
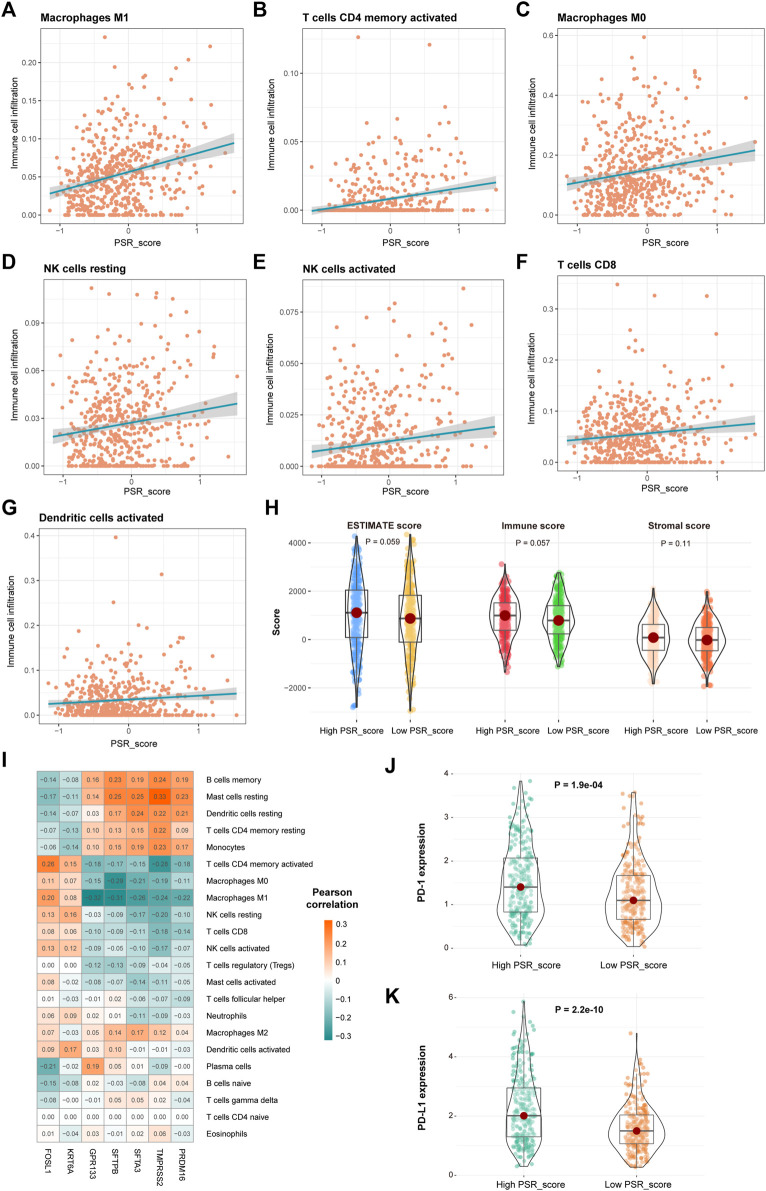
Correlation of PSR_score and immune cell infiltration. **(A–G)** Positive correlation between PRG_score and immune cells. **(H)** Distribution of the ESTIMATE score in high and low PRG_score groups. **(I)** Correlations between the abundance of immune cells and seven genes in the proposed model. **(J–K)** Expression of PD-1 and PD-L1 in high and low PRG_score groups.

Pearson correlation analysis was performed to assess the relationship between PRG_score and the number of immune cells to study the link between PRG_score and immunological infiltration. Infiltration of “macrophages M1,” “T cells CD4 memory activated,” “macrophages M0,” “NK cells resting,” “NK cells activated,” “T cells CD8,” and “dendritic cells activated” was significantly positively connected with PRG_score (Figure 6A-G, *p* < 0.05, Pearson correlation analysis). Furthermore, ESTIMATE score of high PRG_score samples was higher than that of low PRG_score samples (Figure 6H). We also investigated the relationship between the expression of seven genes in the model and immune cells. We discovered that the quantity of most immune cells was associated with the expression of these genes (Figure 6I). In TCGA LUAD cohorts, the expression of PD-1 and PD-L1 was significantly higher in high PRG_score samples than in low PRG_score samples (Figures 6J,K, *p* < 0.05).

**FIGURE 7:**
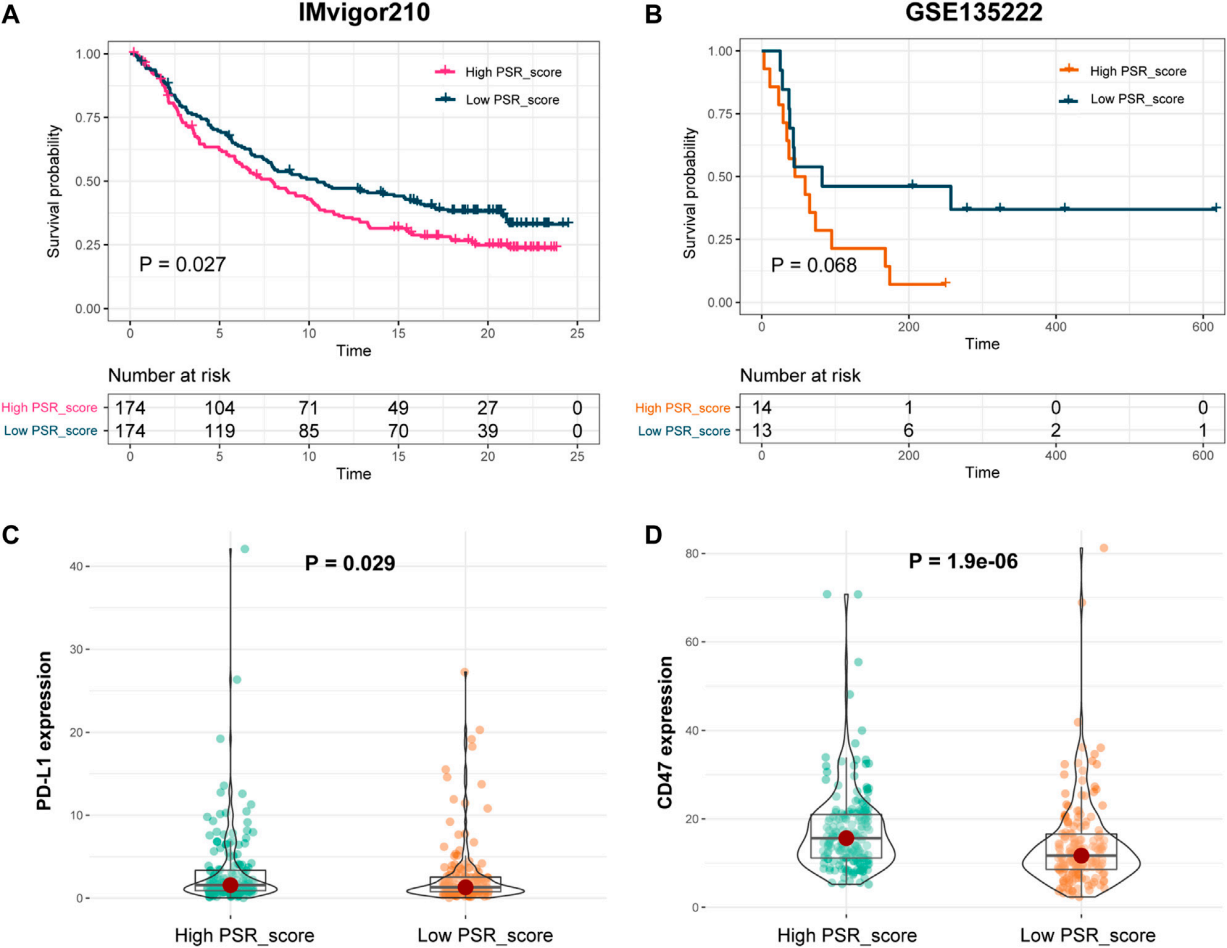
Prognosis power of PSR_score in patients with immunotherapy. **(A–B)** Log-rank test was used to assess the difference in OS between high and low PSR_score samples in IMvigor210 and GSE135222 cohorts. **(C–D)** Expression of PD-L1 and CD47 in high and low PRG_score groups in the IMvigor210 cohort.

To further explore if the risk score can predict patients' responses to immunotherapy, we compared OS of patients with a high PRG_score versus low PRG_score who were receiving immunotherapy. In IMvigor210 and GSE135222 cohorts, patients with a high PRG_score had a significantly worse prognosis (Figures 7A,B, *p* < 0.05, log-rank test). In addition, we looked at the differences in immune checkpoint gene expression between high and low PRG_score groups. PD-L1 and CD47 in the high PRG_score group of the IMvigor210 cohort were significantly greater than those in the low PRG_score group (Figures 7C,D; *p* < 0.05).

## Discussion

Increasing research has proven the role of pyroptosis in cancer progression (Xia et al., 2019; Fang et al., 2020; Tan et al., 2021). However, the prognostic potential of pyroptosis in LUAD is still unknown. The genetic and transcriptional mutations of PRGs in LUAD were detected in this study. TP53 had the highest mutation frequency among the mutated genes (Figure 1). TP53 mutation was linked to the downregulation of PRGs such as CHMP7, IRF2, CASP4, ELANE, BAX, and TIRAP (Supplementary Figure S1). By elevating the pyroptotic level, the transcription factor p53 may be able to suppress lung cancer cell proliferation (Braden et al., 2014; Zhang et al., 2019). In LUAD samples, 30 PRGs were differentially expressed (Figure 1). Based on the 30 DEGs, the patients were divided into two groups. The OS of subtype 1 is higher than that of subtype 2 (Figure 2).

The score of 25 therapeutic signature sets was calculated to investigate the therapeutic differentiation between the two subtypes. There were 23 therapeutic signatures that differed between the two subtypes. Patients with subtype 2 responded well to the treatment (Figures 3A,B). The difference in TME between the two subtypes was then examined. Compared to subtype 1, subtype 2 had high immunological infiltration of M1 macrophages, NK cells, CD4^+^, and CD8^+^ T cells. Patients with LUAD have lower numbers of NK cells, CD4^+^, and CD8^+^ T cells (Cui et al., 2021). CD4^+^ and CD8^+^ T cells are critical in mediating antitumor responses. Patients with higher numbers of CD4^+^ T cells respond better to PD-1 blockade therapy (Kagamu et al., 2020). The samples showed greater levels of immune infiltration in subtype 2. As a result, the variation in immune statuses may cause a differential prognosis between the two subtypes.

Following that, DEGs between subtypes 1 and 2 were identified. A total of 719 DEGs were found to be enriched in immune-related pathways and processes, such as “leukocyte activation.” A co-expression network was constructed by WGCNA using these DEGs, and four modules were identified. The blue module was associated with the prognosis and tumor stage (Figure 4).

A seven-gene-involved prognosis model was created using LASSO Cox regression to investigate the prognostic value of genes in the blue module, comprising FOSL1, KRT6A, GPR133, TMPRSS2, PRDM16, SFTPB, and SFTA3. The patients were divided into two groups based on the prognostic model: those with a high PSR_score and those with a low PSR_score. Patients in the low PSR_score group have a better OS than those in the high PSR_score group in TCGA cohort. The GEO cohorts yielded comparable results (Figure 5). FOSL1 and GPR133 were investigated for their roles in LUAD. FOSL1 expression, for example, was found to be inversely associated with the OS of lung cancer patients, particularly those with LUAD. FOSL1 induction might enhance LUAD initiation, whereas FOSL1 deficiency inhibits LUAD cell proliferation and promotes apoptosis (Elangovan et al., 2018). The GPR133 levels were found to be lower in LUAD samples. Higher GPR133 expression was associated with a better prognosis in LUAD patients. Increased GPR133 expression in LUAD patients may limit cell proliferation and tumor progression (Wu et al., 2021).

Then, the correlation between PSR_score and cancer immune features was evaluated. M1 and M0 macrophages, CD4^+^ and CD8^+^ T cells, and NK cells were all found to be positively linked with the PRG scores. Higher levels of immunological infection were associated with higher PRG scores and ESTIMATE scores. The infection levels of B cells, CD4^+^ T cells, and neutrophils have prognostic values for LUAD (Kadara et al., 2017; Ma et al., 2020; Zhang and Ma, 2021). Furthermore, in the TCGA LUAD cohort, patients in the high PRG_score group have higher expression levels of PD-1 and PD-L1 than those in the low PRG_score group (Figure 6). According to the findings, an increased PD-1 and PD-L1 expression was associated with a poor prognosis in LUAD patients (Teglasi et al., 2017; Xia et al., 2017).

Finally, we investigated the predictive value of PSR_score for immunotherapy response. Patients with a low PRG_score have a greater OS rate than those with a high PRG_score. Furthermore, in the IMvigor210 cohort, PD-L1 and CD47 were strongly expressed in the high PSR_score group (Figure 7). LUAD TME was a good predictor of response to immune checkpoint blockade treatment (Wang et al., 2020; Yi et al., 2021). These findings suggested that LUAD patients with a high PSR_score had a poor prognosis due to TME. As a result, the pyroptosis-associated model developed shows predictive potential for responsiveness to immune checkpoint blockade in LUAD. Our results investigated the role of pyroptosis in TME remodeling. Using PRGs, we found a subtype with a poor prognosis, which provides new insights into locating possible immunotherapy manufacturers.

## Conclusion

The current study established a pyroptosis-related signature for predicting OS and immunotherapy responses in LUAD, which may lead to new insights into the individualized LUAD therapy.

## Data Availability

The original contributions presented in the study are included in the article/Supplementary Material. Further inquiries can be directed to the corresponding author.
