# Supplementary material for: A Pyroptosis-Related Signature Predicts Overall Survival and Immunotherapy Responses in Lung Adenocarcinoma
Source: Front Genet. 2022 Jun 20;13:891301. doi: 10.3389/fgene.2022.891301 (PMC9252528; doi:10.3389/fgene.2022.891301)
Supplement: Supplementary file 2 [file Table1.DOCX]

**Table S1. Baseline characteristics of patients in TCGA LUAD cohort**

| **Characteristics** | **Whole cohort** | **High PSR_score** | **Low PSR_score** | ***p*** |
| --- | --- | --- | --- | --- |
| TCGA cohort | (n=510) | (n=255) | (n=255) |  |
| Gender |  |  |  | 0.18 |
| Male | 235(46.08%) | 125(49.02%) | 110(43.14%) |  |
| Female | 275(53.92%) | 130(50.98%) | 145(56.86%) |  |
| Age |  |  |  | 0.19 |
| <65 years | 219(42.94%) | 116(45.49%) | 103(40.39%) |  |
| >=65 years | 272(53.33%) | 128(50.2%) | 144(56.47%) |  |
| T-stage |  |  |  | 0.056 |
| T1 | 167(32.75%) | 70(27.45%) | 97(38.04%) |  |
| T2 | 276(54.12%) | 146(57.25%) | 130(50.98%) |  |
| T3 | 45(8.82%) | 27(10.59%) | 18(7.06%) |  |
| T4 | 19(3.73%) | 11(4.31%) | 8(3.14%) |  |
| N-stage |  |  |  | 5.3e-06 |
| N0 | 327(64.12%) | 139(54.51%) | 188(73.73%) |  |
| N1 | 95(18.63%) | 64(25.1%) | 31(12.16%) |  |
| N2 | 74(14.51%) | 48(18.82%) | 26(10.2%) |  |
| N3 | 2(0.39%) | 2(0.78%) | 0(0%) |  |
| M-stage |  |  |  | 0.31 |
| M0 | 343(67.25%) | 170(66.67%) | 173(67.84%) |  |
| M1 | 25(4.9%) | 15(5.88%) | 10(3.92%) |  |
| Stage |  |  |  | 0.00010 |
| I | 273(53.53%) | 112(43.92%) | 161(63.14%) |  |
| II | 120(23.53%) | 72(28.24%) | 48(18.82%) |  |
| III | 84(16.47%) | 54(21.18%) | 30(11.76%) |  |
| IV | 26(5.1%) | 15(5.88%) | 11(4.31%) |  |
