# Supplementary figures and images for: A Pyroptosis-Related Signature Predicts Overall Survival and Immunotherapy Responses in Lung Adenocarcinoma
Source: Front Genet. 2022 Jun 20;13:891301. doi: 10.3389/fgene.2022.891301 (PMC9252528; doi:10.3389/fgene.2022.891301)

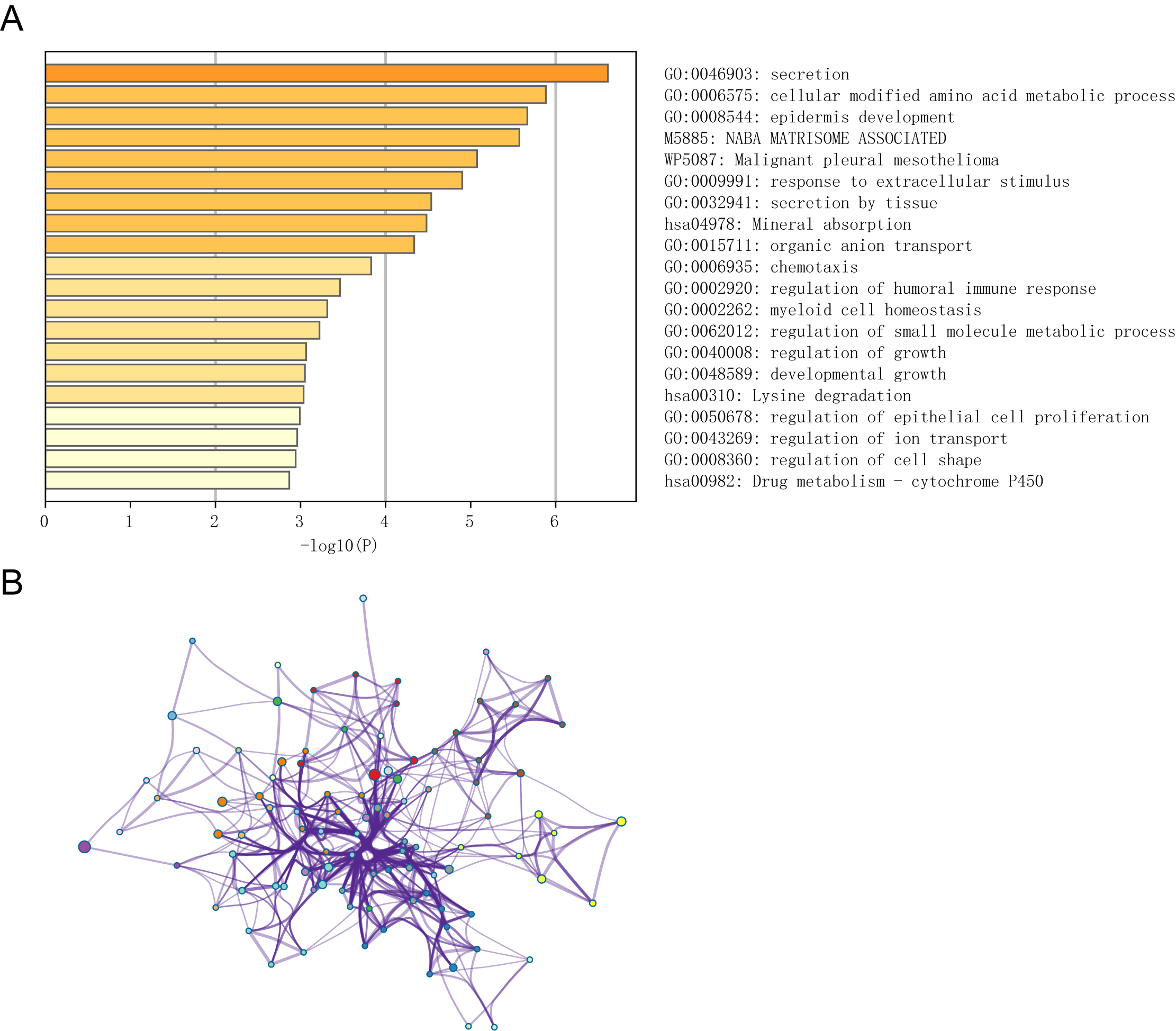

Supplement: Supplementary file 3 [file Image3.TIF]

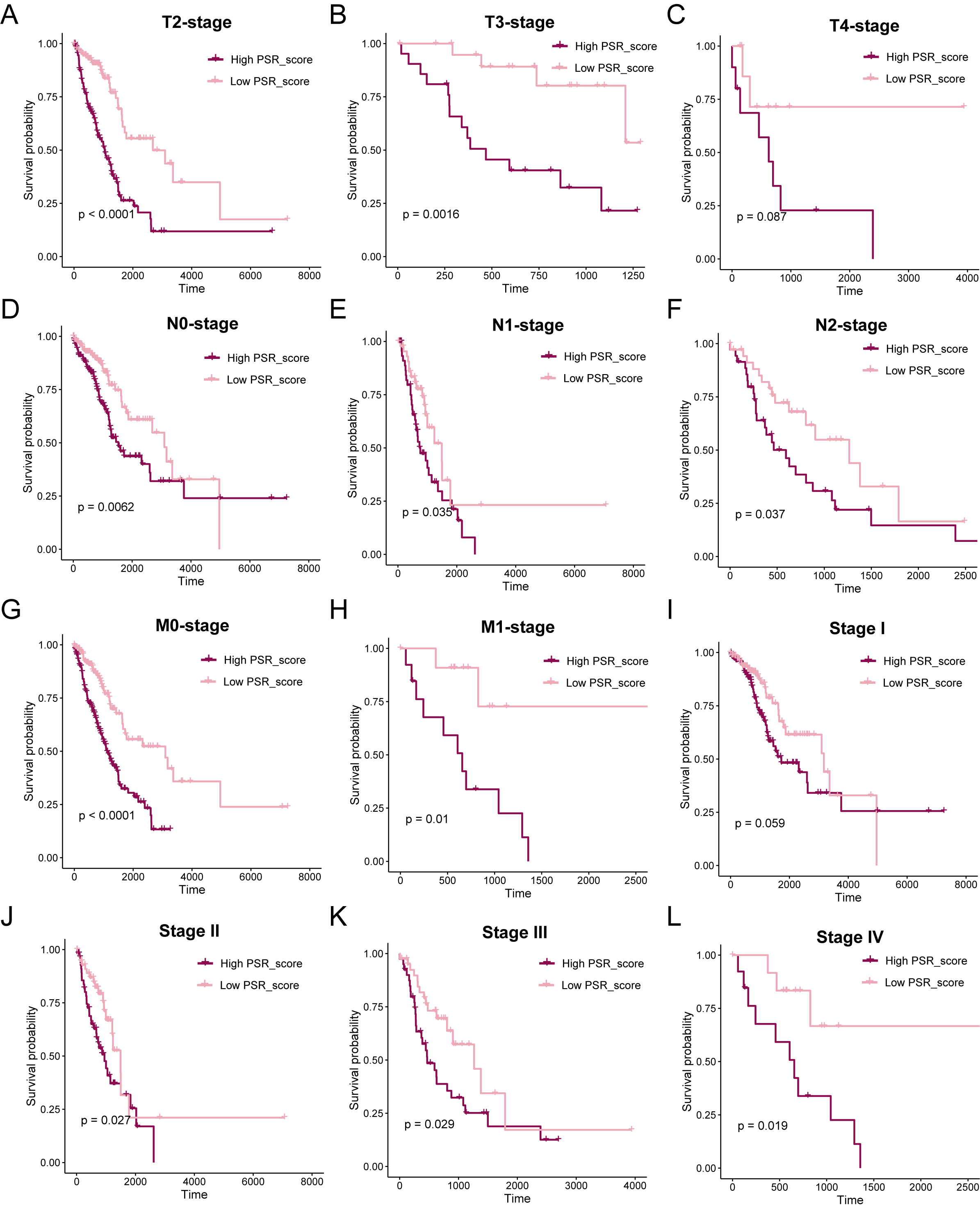

Supplement: Supplementary file 4 [file Image4.TIF]

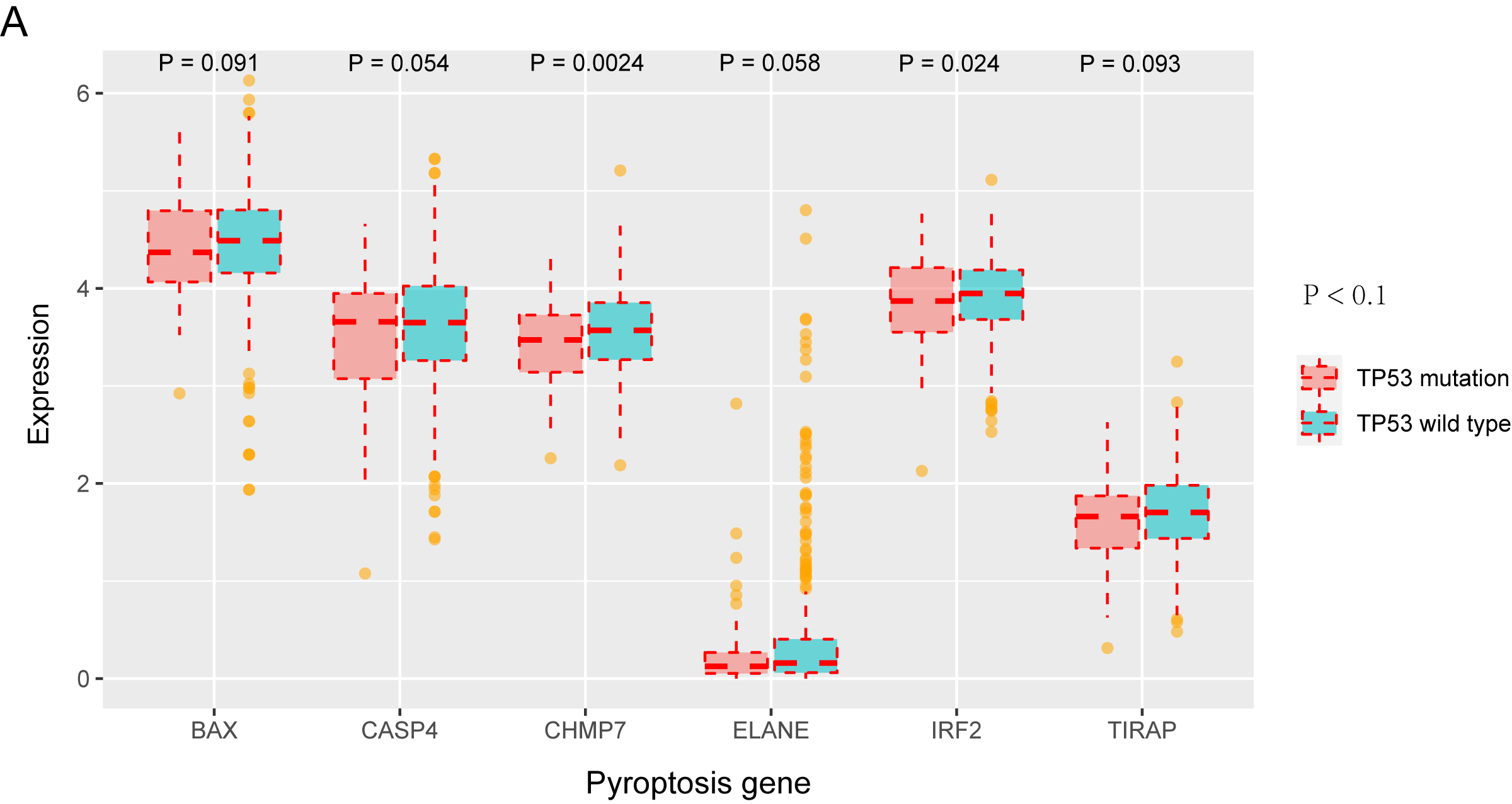

Supplement: Supplementary file 5 [file Image2.TIF]

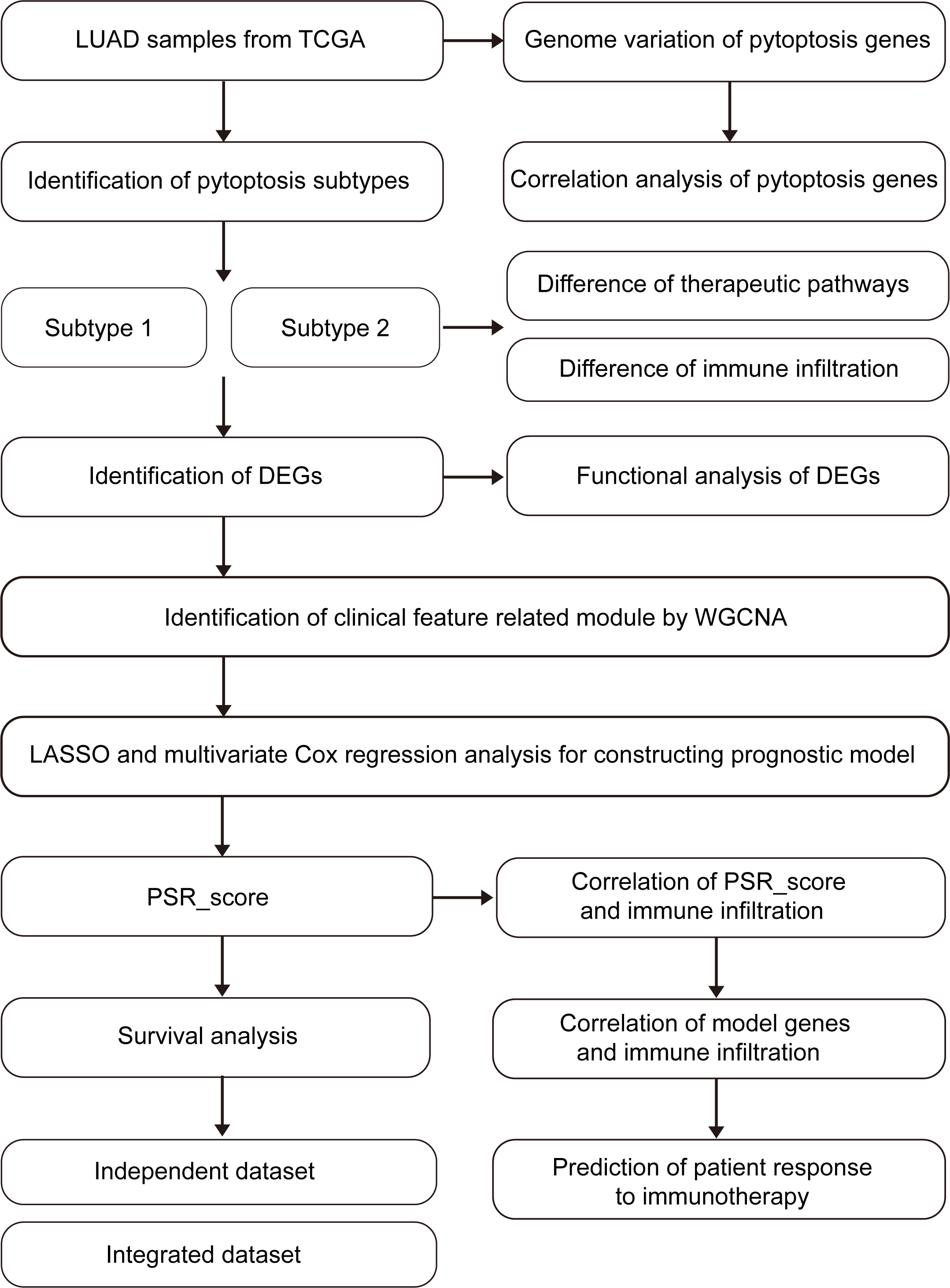

Supplement: Supplementary file 6 [file Image1.TIF]

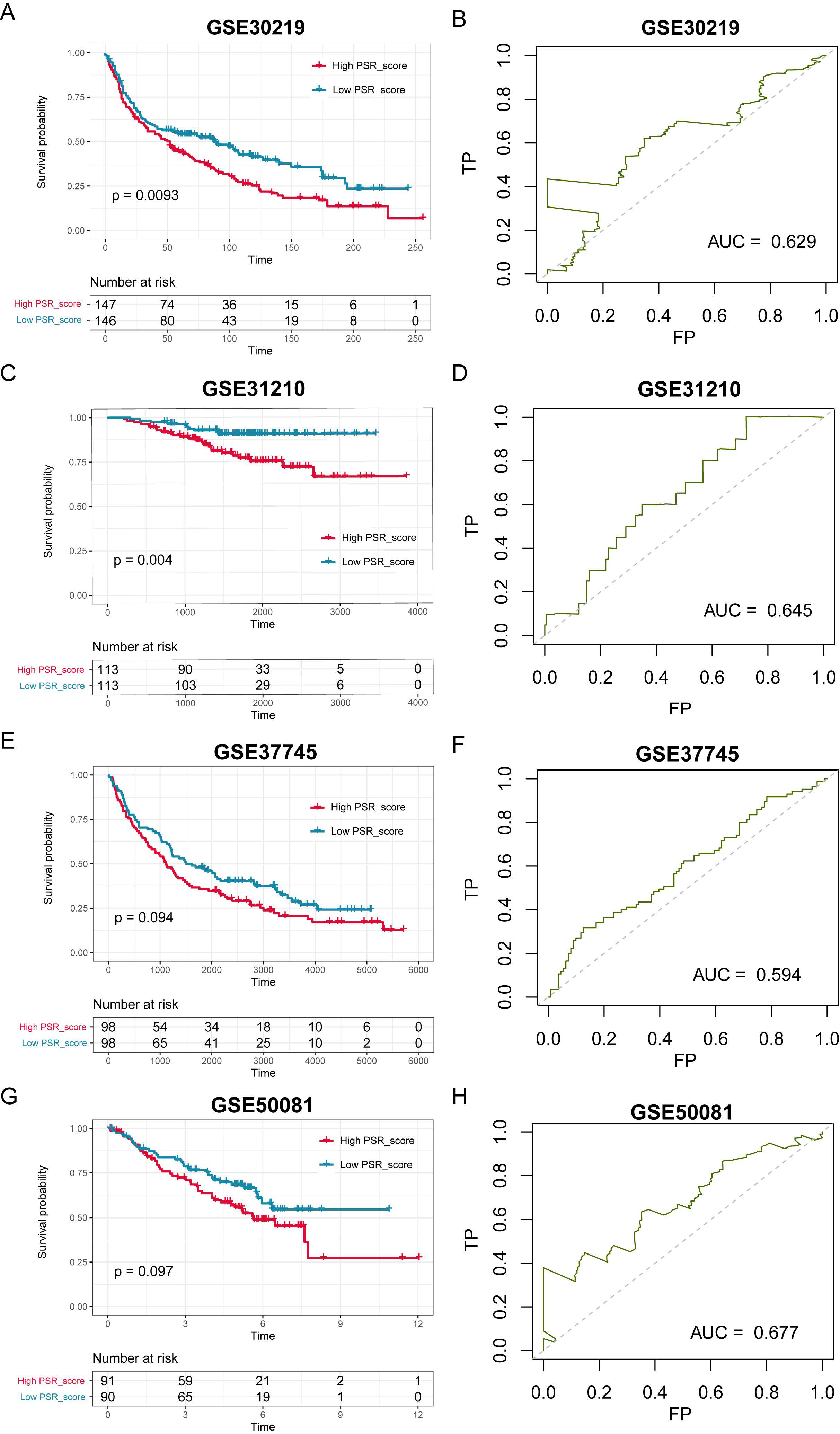

Supplement: Supplementary file 9 [file Image5.TIF]
