# Supplementary material for: A Pyroptosis-Related Signature Predicts Overall Survival and Immunotherapy Responses in Lung Adenocarcinoma
Source: Front Genet. 2022 Jun 20;13:891301. doi: 10.3389/fgene.2022.891301 (PMC9252528; doi:10.3389/fgene.2022.891301)
Supplement: Supplementary file 7 [file Table2.DOCX]

**Table S2. Baseline characteristics of patients in GEO cohorts**

| **Characteristics** | **Whole cohort** | **High PSR_score** | **Low PSR_score** | ***p*** |
| --- | --- | --- | --- | --- |
| **GSE30219** | (n=293) | (n=146) | (n=146) |  |
| Gender |  |  |  | 0.032 |
| Male | 250(85.32%) | 131(89.73%) | 118(80.82%) |  |
| Female | 43(14.68%) | 15(10.27%) | 28(19.18%) |  |
| Age |  |  |  | 0.14 |
| <65 years | 166(56.66%) | 77(52.74%) | 89(60.96%) |  |
| >=65 years | 126(43%) | 69(47.26%) | 56(38.36%) |  |
| T-stage |  |  |  | 0.23 |
| T1 | 166(56.66%) | 80(54.79%) | 86(58.9%) |  |
| T2 | 69(23.55%) | 32(21.92%) | 36(24.66%) |  |
| T3 | 31(10.58%) | 19(13.01%) | 12(8.22%) |  |
| T4 | 21(7.17%) | 14(9.59%) | 7(4.79%) |  |
| N-stage |  |  |  | 0.094 |
| N0 | 198(67.58%) | 94(64.38%) | 103(70.55%) |  |
| N1 | 53(18.09%) | 34(23.29%) | 19(13.01%) |  |
| N2 | 30(10.24%) | 14(9.59%) | 16(10.96%) |  |
| N3 | 10(3.41%) | 3(2.05%) | 7(4.79%) |  |
| M-stage |  |  |  | 0.99 |
| M0 | 282(96.25%) | 140(95.89%) | 141(96.58%) |  |
| M1 | 8(2.73%) | 4(2.74%) | 4(2.74%) |  |
|  | | | | |
| **Characteristics** | **Whole cohort** | **High PSR_score** | **Low PSR_score** | ***p*** |
| **GSE31210** | (n=226) | (n=113) | (n=113) |  |
| Gender |  |  |  | 0.0022 |
| Male | 105(46.46%) | 64(56.64%) | 41(36.28%) |  |
| Female | 121(53.54%) | 49(43.36%) | 72(63.72%) |  |
| Age |  |  |  | 0.77 |
| <65 years | 164(72.57%) | 81(71.68%) | 83(73.45%) |  |
| >=65 years | 62(27.43%) | 32(28.32%) | 30(26.55%) |  |
| Stage |  |  |  | 7.5e-05 |
| Stage I | 168(72.57%) | 71(71.68%) | 97(73.45%) |  |
| Stage II | 58(27.43%) | 42(28.32%) | 16(26.55%) |  |
| Smoking status |  |  |  | 0.0052 |
| Ever-smoker | 111(49.12%) | 66(58.41%) | 45(39.82%) |  |
| Never-smoker | 115(50.88%) | 47(41.59%) | 68(60.18%) |  |
|  | | | | |
| **Characteristics** | **Whole cohort** | **High PSR_score** | **Low PSR_score** | ***p*** |
| **GSE37745** | (n=196) | (n=98) | (n=98) |  |
| Gender |  |  |  | 0.00034 |
| Male | 107(54.59%) | 66(67.35%) | 41(41.84%) |  |
| Female | 89(45.41%) | 32(32.65%) | 57(58.16%) |  |
| Age |  |  |  | 0.086 |
| <65 years | 94(47.96%) | 41(41.84%) | 53(54.08%) |  |
| >=65 years | 102(52.04%) | 57(58.16%) | 45(45.92%) |  |
| Stage |  |  |  | 0.60 |
| I | 130(66.33%) | 63(64.29%) | 67(68.37%) |  |
| II | 35(17.86%) | 20(20.41%) | 15(15.31%) |  |
| III | 27(13.78%) | 14(14.29%) | 13(13.27%) |  |
| IV | 4(2.04%) | 1(1.02%) | 3(3.06%) |  |
| Histology |  |  |  | <2.2e-16 |
| adeno | 106(54.08%) | 18(18.37%) | 88(89.8%) |  |
| large | 24(12.24%) | 15(20.41%) | 9(15.31%) |  |
| squamous | 66(33.67%) | 65(66.33%) | 1(1.02%) |  |
|  | | | | |
| **Characteristics** | **Whole cohort** | **High PSR_score** | **Low PSR_score** | ***p*** |
| **GSE50081** | (n=181) | (n=91) | (n=90) |  |
| Gender |  |  |  | 0.42 |
| Male | 98(54.14%) | 52(57.14%) | 46(51.11%) |  |
| Female | 83(45.86%) | 39(42.86%) | 44(48.89%) |  |
| Age |  |  |  | 0.40 |
| <65 years | 59(32.6%) | 27(29.67%) | 32(35.56%) |  |
| >=65 years | 122(67.4%) | 64(70.33%) | 58(64.44%) |  |
| T-stage |  |  |  | 0.0057 |
| T1 | 57(31.49%) | 20(21.98%) | 37(41.11%) |  |
| T2 | 122(67.4%) | 71(70.33%) | 51(64.44%) |  |
| T3 | 2(1.1%) | 0(0%) | 2(2.22%) |  |
| N-stage |  |  |  | 0.11 |
| N0 | 129(71.27%) | 60(65.93%) | 69(76.67%) |  |
| N1 | 52(28.73%) | 31(34.07%) | 21(23.33%) |  |
| Stage |  |  |  | 0.21 |
| I | 127(70.17%) | 60(65.93%) | 67(74.44%) |  |
| II | 54(29.83%) | 31(34.07%) | 23(25.56%) |  |
